# Stillbirth as left truncation for early neonatal death in California, 1989–2015: a time-series study

**DOI:** 10.1186/s12884-021-03852-z

**Published:** 2021-07-02

**Authors:** Tim A. Bruckner, Samantha Gailey, Abhery Das, Alison Gemmill, Joan A. Casey, Ralph Catalano, Gary M. Shaw, Jennifer Zeitlin

**Affiliations:** 1 Program in Public Health & Center for Population, Inequality, and Policy, University of California Irvine, 653 E. Peltason Dr., Irvine, CA 92697 USA; 2 School of Social Ecology, University of California Irvine, 209 Social Ecology I, Irvine, CA 92697 USA; 3 Program in Public Health, University of California Irvine, 653 E. Peltason Dr., Irvine, CA 92697 USA; 4 Population, Family and Reproductive Health, Johns Hopkins Bloomberg School of Public Health, 615 N. Wolfe St., Baltimore, MD 21205 USA; 5 Mailman School of Public Health, Columbia University, 722 W. 168th St., New York, NY 10032 USA; 6 School of Public Health, University of California Berkeley, Berkeley, CA 94720 USA; 7 School of Medicine, Stanford University, Stanford, CA 94305 USA; 8 Université de Paris, CRESS, Obstetrical, Perinatal and Pediatric Epidemiology Research Team, EPOPé, INSERM, INRA, F-75004 Paris, France

**Keywords:** Stillbirth, Neonatal death, Live birth, Left truncation Bias

## Abstract

**Background:**

Some scholars posit that attempts to avert stillbirth among extremely preterm gestations may result in a live birth but an early neonatal death. The literature, however, reports no empirical test of this potential form of left truncation. We examine whether annual cohorts delivered at extremely preterm gestational ages show an inverse correlation between their incidence of stillbirth and early neonatal death.

**Methods:**

We retrieved live birth and infant death information from the California Linked Birth and Infant Death Cohort Files for years 1989 to 2015. We defined the extremely preterm period as delivery from 22 to < 28 weeks of gestation and early neonatal death as infant death at less than 7 days of life. We calculated proportions of stillbirth and early neonatal death separately by cohort year, race/ethnicity, and sex. Our correlational analysis controlled for well-documented declines in neonatal mortality over time.

**Results:**

California reported 89,276 extremely preterm deliveries (live births and stillbirths) to Hispanic, non-Hispanic (NH) Black, and NH white mothers from 1989 to 2015. Findings indicate an inverse correlation between stillbirth and early neonatal death in the same cohort year (coefficient: -0.27, 95% CI of − 0.11; − 0.42). Results remain robust to alternative specifications and falsification tests.

**Conclusions:**

Findings support the notion that cohorts with an elevated risk of stillbirth also show a reduced risk of early neonatal death among extremely preterm deliveries. Results add to the evidence base that selection *in utero* may influence the survival characteristics of live-born cohorts.

**Supplementary Information:**

The online version contains supplementary material available at 10.1186/s12884-021-03852-z.

## Background

Infants born alive at extremely early gestational ages face substantial risk of imminent death. Extremely preterm births (i.e., delivery at less than 28 weeks' gestational age [GA]), for instance, account for less than 1% of all live births but over 40% of neonatal mortality [1, 2]. The majority of these infant deaths occur in the early neonatal period which extends to less than 7 days after birth. Substantial advancements in obstetric monitoring, neonatal care, and medical technology since the 1980s in high-income countries correspond with reductions over time in neonatal mortality [2, 3]. However, the incidence of early neonatal mortality among extremely preterm live births in the US remains between 15 and 20% [4, 5].

Epidemiologists continue to debate how to best estimate the population at risk in the perinatal period [6–11]. Some argue that all fetuses which pass through a particular GA “starting point” (e.g., > 22 weeks) represent a risk set, or denominator, of gestations at risk of ending with a neonatal death. According to this reasoning, those that die *in utero* and receive a classification of stillbirth would also appear in this risk set. This logic appears reflected in the rationale for the use of a composite outcome of perinatal death in randomized trials of obstetrical interventions in which both stillbirths and early neonatal deaths represent “cases” [6, 12]. This composite perinatal death outcome coheres with the argument that stillbirths near week 22, at the threshold of viability, would have been at elevated risk for early neonatal death had they been born live. This “left truncation” argument [13, 14], if distilled to the realm of clinical decision-making, assumes that attempts to avert imminent stillbirth among threatened gestations may “convert” a subset of them to a live-born delivery but result in an early neonatal death.

The literature, however, also includes reports in which stillbirth and early neonatal death may be considered as distinct entities [7, 15]. The argument arises from two strands of evidence. First, in high-income countries that use consistent definitions and classification schemes, risk factors differ for stillbirth and early neonatal death. For instance, in the 1990s in Canada, congenital anomalies reportedly accounted for over 45% of early neonatal deaths but only ~ 9% of stillbirths reaching 25 weeks' GA [15]. Second, the risk of early neonatal death in the US has fallen, but the risk of stillbirth at GAs in the extremely preterm period (< 28 weeks' GA) has remained unchanged [16]. These divergent population-level patterns indicate distinct antecedents of early neonatal death and stillbirth among extremely preterm gestations. Taken together, the field continues to debate various circumstances under which researchers should regard stillbirth and early neonatal death as joint or distinct outcomes [11, 17].

A recent report using data from California finds that an abrupt downward shift in stillbirths over time coincides with an upward shift in live births delivered in the extremely preterm period [18]. This report, while suggestive of left truncation, has no information on infant death and therefore cannot address whether early neonatal death in the extremely preterm period falls in pregnancy cohorts in which the risk of stillbirth rises. Understanding this potential relation would inform the extent to which intensity of fetal selection shapes the survival characteristics of live-born cohorts. In this paper, we contribute to the literature by testing in California over a 26-year period whether annual cohorts delivered at extremely preterm GAs show an inverse correlation between their proportions of stillbirth and early neonatal death.

We apply methods that adjust for the secular decline since 1990 in the risk of early neonatal death before conducting our correlational test. In addition, given well-documented differences in risks of stillbirth and early neonatal death by race/ethnicity and fetal sex, we stratify pregnancy cohorts by maternal race/ethnicity and sex of gestation [15, 16, 19–23]. Our analysis focuses on California because, in addition to accounting for ~ 15% of all US births, the state uses consistent definitions and data collection practices for recording stillbirths over a long time period (i.e., 1989–2015) [24]. The US file, by contrast, makes available fewer years of cohort mortality data and reflects a mix of states with substantial differences in quality and reporting practices of stillbirth.

## Methods

We retrieved live birth and infant death information from the California Linked Birth and Infant Death Cohort File (BCF) from 1989 to 2015. The cohort nature of the BCF allows for the estimation of incident early neonatal death. Our study period began in 1989 and ended in 2015. This time period uses consistent definitions for live births and early neonatal deaths. The methodology of reporting births and infant deaths in California has not changed over the time period and remains nearly 100% complete [22, 24]. For administrative reasons, the California Department of Health Services did not create a BCF for 1998. As a result, we did not include the 1998 birth cohort in our analysis. The institutional review boards at the California Department of Public Health (# 2018–065) and the University of California, Irvine (# 2013–9716) approved the use of these data for our study.

We retrieved stillbirth information from the California Fetal Death file. The State of California defines a stillbirth as a “death prior to the complete expulsion or extraction from its mother of a product of human conception. The death is indicated by the fact that the fetus does not breathe or show any other evidence of life such as beating of the heart, pulsation of the umbilical cord, or definite movement of voluntary muscles [25].” California’s Health and Safety Code requires reporting of all stillbirths after the 20th week of gestation except for induced abortions [25–28]. The California Department of Health Services uses a standard protocol to perform quality control checks and data processing. We, as with previous research [26], calculated the proportion of stillbirths for each year by dividing the count of stillbirths by the sum of live births and stillbirths among extremely preterm deliveries.

Consistent with the definition from the World Health Organization [29], we specified the extremely preterm period as delivery from 22 weeks 0/7 days to 27 weeks 6/7 days of gestation. Previous literature uses this span of gestational ages to define extreme preterm delivery for two reasons [2, 30, 31]. First, many clinicians argue that 22 0/7 weeks represents the lowest gestational age cutoff for a viable delivery [32, 33]. Second, the risk of infant death among live births beginning at 28 0/7 weeks falls below 5% [34]. We therefore restricted our analysis to stillbirths and live births from 22 0/7 to 27 6/7 weeks of gestation. In addition, we restricted the sample to singleton gestations owing to the shorter mean gestational age of multiple births and the greater risk of perinatal death among them regardless of gestational age [34].

We used early neonatal death as a key indicator of perinatal health whose causes likely originate during pregnancy [35]. Early neonatal death is defined as an infant death at less than 7 days of life [36, 37]. Consistent with definitions used for surveillance in California and elsewhere, only live births represent the risk set of possible early neonatal deaths [35, 38]. We therefore calculated the proportion of early neonatal deaths by dividing the number of infant deaths in the first 7 days of life by the total number of live births.

The risk of stillbirth and early neonatal death vary substantially by race/ethnicity and by sex [16, 39, 40]. For this reason, we arrayed all extremely preterm deliveries by race/ethnicity and sex before conducting the statistical analyses. Given that the notion of left truncation represents a cohort concept, we did not include individual-level controls in our aggregate-level test. We excluded records with missing or unknown race/ethnicity (0.87%) or sex (<.0001%) as well as live birth records with implausible birthweight for gestational age information (1.2%) [41] and stillbirths with missing values for GA (10.2%). The welcomed rarity of extremely preterm deliveries creates an analytic challenge in providing stable estimates, by race/ethnicity and sex, of the annual incidence of stillbirth and early neonatal death. To minimize the role of stochastic variation in our analysis, we focused only on race/ethnicities with a minimum of 100 extremely preterm deliveries per sex in each study year. This restriction yielded three race/ethnicities: non-Hispanic (NH) Black, NH white, and Hispanic.

### Statistical analysis

We first plotted, by race/ethnicity and sex, the annual incidence (1989–2015) of stillbirth and early neonatal death among extremely preterm deliveries. Second, given the well-documented declines over time in perinatal mortality, we removed trend from these series (if detected by a Dickey-Fuller test) by employing ordinary least squares linear regression analysis to fit a year variable (continuous, from 1 to 26, where 1989 = 1, 1990 = 2, etc.) [42]. We removed trend to minimize confounding due to secular improvements in perinatal care over time that could induce a positive correlation between the risk of fetal and early neonatal death. Third, we tested our hypothesized inverse association between yearly risks of fetal and early neonatal death by calculating the Pearson correlation coefficient between the de-trended annual values of the two series. Given the three race/ethnicities, two sexes, and 26 years studied, the operational sample size for the correlational analysis is 156 (i.e., 3 × 2 × 26 = 156).

We conducted additional sensitivity checks including autoregressive, integrated, moving average (ARIMA) time-series analyses if we discovered an inverse correlation. We applied a transfer function within the ARIMA context [43], which identifies and removes patterns from the early neonatal death series before inserting the independent variable (i.e., residual values of stillbirth) into the test equation. ARIMA transfer functions provide more efficient estimation of standard errors than do simple ordinary least squares correlational analyses since they remove autocorrelation. Next, we repeated all analyses but examined the correlation coefficient between stillbirth and neonatal death (i.e., death within first 28 days after birth), rather than early neonatal death, given that a small but non-negligible fraction of frail and extremely preterm infants die between 7 and 28 days of life [4].

## Results

Over the test period, California recorded 89,276 extremely preterm deliveries (live births and stillbirths) to Hispanic, NH Black, and NH white mothers. Table 1 describes the annual mean and range of live births, stillbirths, and early neonatal deaths by race/ethnicity. The crude incidence of early neonatal death is 20.2 per 100 extremely preterm live births. NH whites show the greatest incidence of neonatal death (20.9 per 100 extremely preterm live births). Table 2 describes the sociodemographic characteristics of the study population.

**Table 1:** Annual mean and range of live births, stillbirths, and early neonatal deaths delivered extremely preterm (22 to 27 weeks of gestational age), by race/ethnicity, in California, 1989 to 2015

	N	Annual mean (SD)^**a**^	Annual range^**b**^
Non-Hispanic Black			
Live births	12,604	485 (89)	377–667
Fetal deaths	2911	112 (18)	82–147
Early neonatal deaths	2525	97 (29)	65–158
Non-Hispanic white			
Live births	21,725	836 (148)	632–1172
Fetal deaths	6602	254 (67)	155–409
Early neonatal deaths	4540	175 (60)	102–328
Hispanic			
Live births	36,058	1387 (178)	972–1760
Fetal deaths	9376	361 (36)	253–433
Early neonatal deaths	7133	274 (35)	224–341

*Abbreviation: SD* standard deviation

^a^Data for 1998 not available

^b^Values rounded to nearest integer

**Table 2:** Maternal and pregnancy characteristics among extremely preterm deliveries (22 to 27 weeks of gestational age) in California, 1989 to 2015

	N^**a**^	%^**b**^
Maternal age		
18 or younger	4736	5.3
18 to 24	27,082	30.3
25 to 29	21,444	24.0
30 to 34	19,641	22.0
35 or older	16,114	18.0
Maternal education		
Less than high school	30,637	34.3
High school graduate	27,222	30.5
Some college	23,065	25.8
College graduate	4760	5.3
Maternal race/ethnicity		
Non-Hispanic Black	16,251	18.2
Non-Hispanic white	25,472	28.5
Hispanic	47,553	53.3
Expected source of payment		
Medicaid	43,054	48.2
Private insurance	34,638	38.8
Other	11,575	13.0
Fetal sex		
Male	47,090	52.7
Female	42,186	47.3

^a^Values from 1998 not available

^b^Column percentages may not sum to 100 due to missing values for that variable

**Fig. 1:**
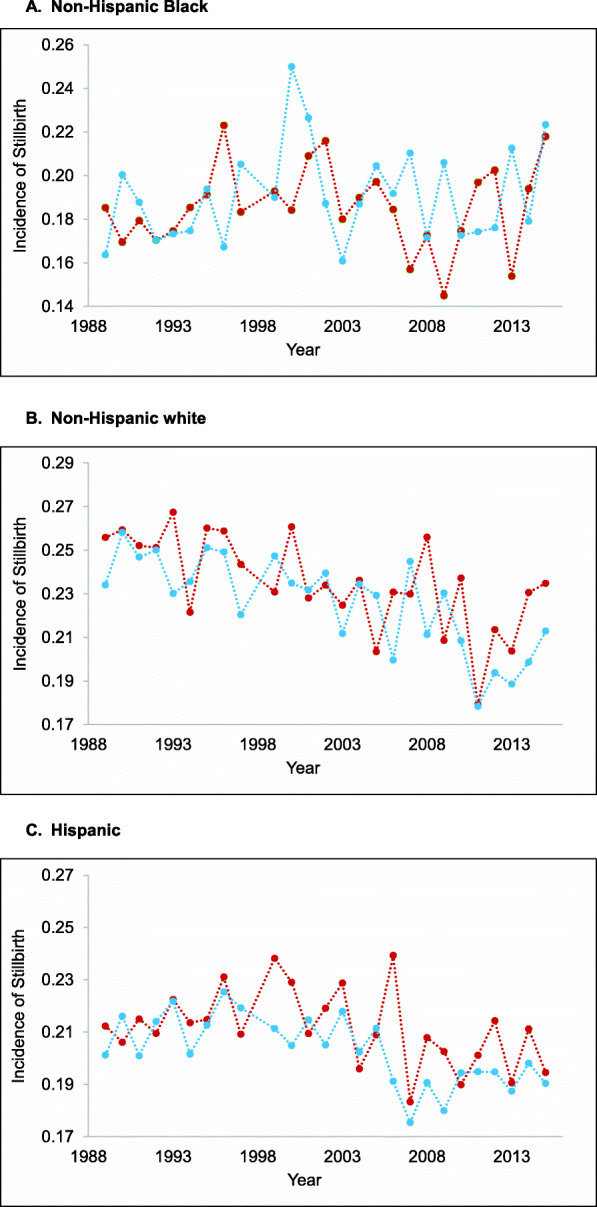
Incidence of stillbirth among extremely preterm deliveries for females (red) and males (blue), by race/ethnicity, in California, 1989 to 2015. **a** Non-Hispanic Black; **b** Non-Hispanic white; **c** Hispanic

Figure 1 a through c plot, by race/ethnicity and sex, the annual stillbirths among extremely preterm deliveries. We caution against comparing mean levels of stillbirth across the panels given that missing GA occurs disproportionately among racial/ethnic minorities and therefore underestimates stillbirth especially among NH Blacks. The plots, rather, are useful in highlighting (within each race/ethnicity) the substantial variation over time in stillbirth. For NH whites, stillbirths show a downward trend over time. For Hispanics, stillbirths also decline over time, but this decline begins with a downward shift in 2007. By contrast, the mean level of stillbirths among NH Blacks is not lower in 2010–2015 relative to 1989–1994. For NH whites and Hispanics (but not NH Blacks), the proportion of stillbirths among females is greater than that of males.

**Fig. 2:**
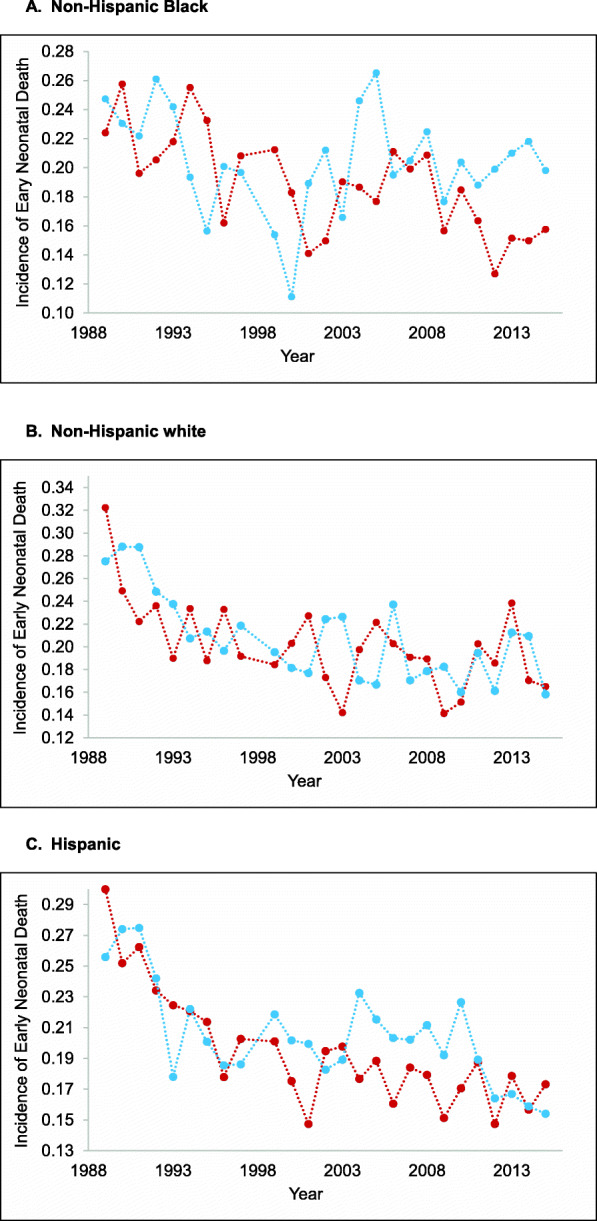
Incidence of early neonatal death among extremely preterm live births for females (red) and males (blue), by race/ethnicity, in California, 1989 to 2015. **a** Non-Hispanic Black; **b** Non-Hispanic white; **c** Hispanic

The risk of early neonatal death declines over time for all race/ethnicities (Fig. 2a through c). Most of this reduction occurs before 2000. After 2000, NH Blacks show a leveling off of early neonatal death, but male risk consistently falls below female risk (i.e., for 13 of the 15 years 2001–2015). This sex-specific pattern, after 2000, in early neonatal death also occurs in Hispanics (i.e., male incidence is less than female incidence for 11 of the 15 years 2001–2015).

**Fig. 3:**
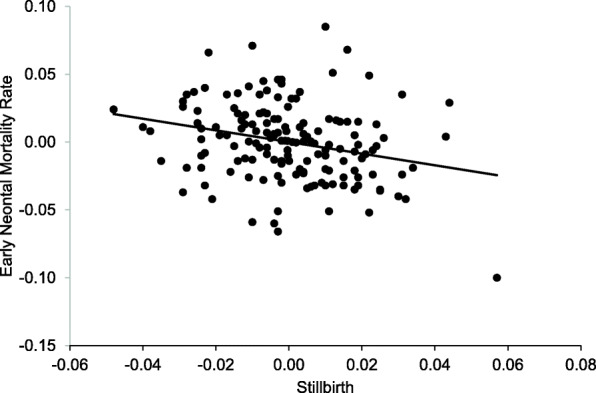
Scatter plot and best fitting line of detrended incidence of stillbirth and early neonatal death among extremely preterm deliveries across 156 race/ethnicity-sex-year cohorts, 1989–2015

The correlation coefficient between the stillbirth and early neonatal death series, after removal of trend, supports left truncation in that it is negative and shows a confidence interval (CI) that does not contain 0 (coefficient.: -0.27, 95% CI of − 0.11; − 0.42). This inverse correlation indicates that, among extremely preterm deliveries, incidence of stillbirth above trend in a particular year corresponds with fewer early neonatal deaths among live births in that year. Figure 3 displays the scatter plot and best fitting line of this inverse correlation.

We conducted an additional sensitivity analysis to assess robustness of findings. We used a transfer function approach within an ARIMA time-series context which proceeded with the following steps. First, we inserted binary indicator variables for each race/ethnicity-gender group to remove mean differences in early neonatal death. Second, we used autocorrelation and partial autocorrelation function routines (as outlined by Box and Jenkins) to identify and remove patterns from the early neonatal death series (i.e., dependent variable) [44]. Patterns detected by these routines include secular trends, cycles, oscillations, and the tendency for high or low values to be “remembered” in subsequent time periods [44]. The residuals of each of the time series (after ARIMA routines) show no patterns, have a mean of 0, and have values statistically independent of one another (Additional file 1: Tables S1 and S2). Third, we inserted the unpatterned values of the stillbirth series (i.e., independent variable) into the test equation and estimated its relation with early neonatal death. ARIMA results show that a 1-unit change in stillbirth varies inversely with a 0.40-unit change in early neonatal death (coefficient: -0.40, 95% CI of − 0.16; − 0.63). Note that the scale of this ARIMA coefficient differs from that of the original test, thus precluding direct comparisons of their magnitude. In addition, we remind the reader that although ARIMA time-series routines increase the efficiency of estimates and rule out confounding by autocorrelation, they are conservative in that they remove patterns from both series without consideration of whether one series (e.g., stillbirth) may have induced a pattern in the other series (e.g., early neonatal death).

As a falsification check we inspected whether the lead and lag cross-correlation coefficients (i.e., stillbirths in year t-1 and early neonatal deaths in year t, and stillbirths in year t + 1 and early neonatal deaths in year t) differ from 0 [45]. These lead and lag tests show no detectable difference from 0 (Table 3). Findings indicate that the discovered inverse association appears specific to pregnancy cohorts which share the same year of delivery.

**Table 3:** Cross-correlation coefficients (standard errors in parentheses) of the detrended incidence of stillbirth and early neonatal death among extremely preterm deliveries (22 to 27 weeks of gestational age) in California, 1989 to 2015

Stillbirth precedes early neonatal death by 1 year	Both series in same year	Stillbirth follows early neonatal death by 1 year
−0.07 (0.08)	−0.27 (0.08)	−0.00 (0.08)

Given that some frail extremely preterm live births die between the 7th and 28th day after birth, we then assessed whether results appear similar when using the de-trended incidence of neonatal death (i.e., death within first 28 days after birth) instead of early neonatal death. As with the main findings, we observe an inverse correlation between detrended annual values of fetal and neonatal death. The result (coefficient: -0.33, 95% CI of −0.17; − 0.49) is farther from the null than that of the original test using early neonatal death.

## Discussion

The literature reports no tests of whether, at the population level, stillbirth represents a form of left truncation for the risk of early neonatal death among live births. We used the longest annual time series available to us—in California, from 1989 to 2015—to test among extremely preterm births whether the incidence of early neonatal death varies inversely with the incidence of stillbirth. Results, which control for well-documented secular trend, support the hypothesis. Annual cohorts which experience relatively lower stillbirth in the extreme preterm period also show elevated risk of neonatal death among live births. This finding builds on recent work documenting an inverse relation between stillbirth and live births in the periviable period [18] and further supports that selection *in utero* may affect the infant health profile of live-born cohorts [23].

Strengths of the analysis include the use of a long annual time series in a populous state with a consistent definition, classification, and reporting protocol for stillbirths. Methods also adjust for well-documented secular declines in early neonatal death, which minimizes the risk of confounding due to medico-technological improvements in perinatal care. In addition, our test rules out confounding due to the changing racial/ethnic composition of cohorts over time since we stratified the series by race/ethnicity. Lastly, the fact that we observed an inverse correlation at the synchronous pregnancy cohort—but not between asynchronous cohorts—further minimizes the possibility of results arising due to chance.

Limitations include that stillbirths remain largely under-reported, especially earlier in the series and during the extreme preterm period when the fetus is smaller [25–28, 40]. Whereas the extent of this under-reporting is unknown, this circumstance likely improved substantially over time [46, 47]. Lack of GA reporting for stillbirth also appears more common among NH Blacks, which precludes direct comparison of GA-specific incidence of stillbirth across race/ethnicity [1]. For example, the incidence of all recorded NH Black stillbirths (including missing and non-missing GA) in California is greater than that of NH whites, but exclusion of cases with missing GA reverses this difference. We, however, know of no evidence that vigilance of reporting stillbirth falls in particular race/ethnicities and years when reporting of early neonatal death increases.

Reductions over time in missing/unknown GA in the California Fetal Death File make it challenging to interpret whether any observed reductions in the risk of stillbirth represent true perinatal health improvements. We note, however, that this circumstance—or other clinical or cultural shifts in reporting—are unlikely to drive our results. Findings remain robust to ARIMA time-series methods which removed such patterns in the series before testing the synchronous correlation. We, nevertheless, note substantial shifts over time in obstetrical practice. Deliveries by cesarean section among periviable births, for instance, have increased substantially over our test period (e.g., from 32% [1989–1997] to over 50% [2007 to 2015] of deliveries between 24 to 27 weeks occurring by cesarean section; see Additional File, Fig. S1). The potential influence of these shifts in clinical practice on stillbirths warrant further investigation.

A recent workshop panel from European countries encourages vital statistics agencies to routinely collect clinical data that could classify stillbirths as occurring either before the initiation of labor (i.e., antepartum) or during labor (i.e., intrapartum) [48]. Such information may assist with identifying a subset of stillbirths whose selection *in utero* affects the risk of early neonatal death [15]. California and other US states do not routinely collect this information. In addition, unlike other countries (e.g., France) [49], termination of pregnancies after 22 weeks (due to, for instance, structural anomaly) is rare and not routinely reported in vital statistics [50, 51]. We encourage collection of this and other information on cause of the stillbirth and reason for induction of labor (taken from medical records) so that researchers can better understand the components of these losses as well as their potential influence on neonatal death.

We focused on deliveries in the extremely preterm period. Excess stillbirths may also induce left truncation for neonatal mortality among live births greater than 28 weeks’ GA. Although the risk of neonatal mortality declines substantially with each advancing week of GA [4], we encourage replication and extension of our work beyond the extreme preterm period. We suspect, however, that any discovered “signal” would appear attenuated relative to the inverse correlation we report for the extreme preterm period.

A recent analysis in California of over 11 million births finds a lower-than-expected frequency of spontaneous preterm live births among NH Black males [52]. The Authors speculate that elevated selection *in utero* of NH Black males in particular may contribute to the “missing” number of NH Black males born preterm. Intriguingly, the discovered outlier in Fig. 3 (bottom right corner)—exceptionally high stillbirth but low early neonatal death—occurs among NH Black males in 2000. In addition, exploration among NH Black males indicates that the correlation coefficient for the stillbirth and early neonatal death series (1989–2015) at the synchronous lag is −0.40 (vs. -0.27 for the overall coefficient across all race/ethnicities and sexes). This evidence, albeit post hoc and exploratory, would appear to warrant further inquiry on the role of late selection *in utero* on the risk of neonatal death especially among NH Black males born preterm.

We acknowledge the descriptive nature of our investigation in that we do not identify underlying causes of stillbirth or early neonatal death. We recommend additional work to identify individual-level risk factors presumed to cause either outcome. In addition, given the population-based nature of our investigation, we caution against using findings to infer individual frailty of specific live births who may have been delivered early in efforts to avoid imminent stillbirth. The annual resolution of our cohorts, moreover, indicates that we cannot align pregnancies by estimated month of conception to establish clear temporal order between stillbirth and early neonatal death. Our work, rather, complements other research examining the potential role of selection *in utero* in shaping the survival characteristics of live-born cohorts [2, 11, 23, 53]. We encourage subsequent analyses of cohorts using larger datasets with sufficient counts of fetal and early neonatal death per month to establish such temporal order between loss *in utero* and the risk of death among extremely preterm live births.

## Conclusions

Annual pregnancy cohorts which experience relatively greater stillbirth in the extremely preterm period also show lower risk of early neonatal death among live births. Results, which remain robust to alternative specifications and falsification tests, add to growing evidence that elevated selection *in utero* contributes to improved survival in live-born cohorts.

## Supplementary Information

**Additional file 1: Table S1.** Coefficients (standard errors in parentheses) for the lagged values of the autocorrelation (ACF) and partial autocorrelation (PACF) functions of the residualized value of the incidence of stillbirths (*n* = 126). **Table S2.** Coefficients (standard errors in parentheses) for the lagged values of the autocorrelation (ACF) and partial autocorrelation (PACF) functions of the residualized value of the incidence of early neonatal deaths (*n* = 126). **Fig. S1.** Proportion of C-sections by perviable gestational age (22–27 weeks) in California by three time periods between 1989 and 2015.

## Data Availability

The datasets generated and/or analyzed during the current study are not publicly available to guarantee the anonymity of individuals. Please contact Tim A. Bruckner (Tim.bruckner@uci.edu) to request access to the datasets used in this study.

## Abbreviations

GA: Gestational age
NH: Non-Hispanic
CI: Confidence interval
