# Supplementary material for: Stillbirth as left truncation for early neonatal death in California, 1989–2015: a time-series study
Source: BMC Pregnancy Childbirth. 2021 Jul 2;21:478. doi: 10.1186/s12884-021-03852-z (PMC8252318; doi:10.1186/s12884-021-03852-z)
Supplement: Supplementary file 1 — Additional file 1: Table S1. Coefficients (standard errors in parentheses) for the lagged values of the autocorrelation (ACF) and partial autocorrelation (PACF) functions of the residualized value of the incidence of stillbirths (n = 126). Table S2. Coefficients (standard errors in parentheses) for the lagged values of the autocorrelation (ACF) and partial autocorrelation (PACF) functions of the residualized value of the incidence of early neonatal deaths (n = 126). Fig. S1. Proportion of C-sections by perviable gestational age (22–27 weeks) in California by three time periods between 1989 and 2015. [file 12884_2021_3852_MOESM1_ESM.docx]

**Additional file 1**

**Table S1.** Coefficients (standard errors in parentheses) for the lagged values of the autocorrelation (ACF) and partial autocorrelation (PACF) functions of the residualized value of the incidence of stillbirths (n=126).

| **Lag at month** | **ACF (SE)** | **Ljung-Box**  **Q Statistic**^a^ | **PACF (SE)** |
| --- | --- | --- | --- |
| 1 | -.03 (.08) | .1 | -.03 (.08) |
| 2 | -.04 (.08) | .5 | -.05 (.08) |
| 3 | .04 (.08) | .5 | -.01 (.08) |
| 4 | .01 (.08) | .5 | -.01 (.08) |
| 5 | -.04 (.08) | .5 | .01 (.08) |
| 6 | .05 (.08) | 1.5 | -.08 (.08) |
| 7 | -.03 (.08) | 1.6 | -.03 (.08) |
| 8 | .05 (.08) | 2.2 | .06 (.08) |
| 9 | .08 (.08) | 4.8 | -.13 (.08) |
| 10 | -.03 (.08) | 4.8 | -.00 (.08) |
| 11 | -.17 (.08) | 5.9 | .07 (.08) |
| 12 | -.11 (.08) | 8.9 | .13 (.08) |

^a^ Q-statistic assesses whether a group of overall autocorrelations differs statistically from 0. None of the Q-statistics reject the null of no difference (based on the chi-square distribution with lag-1 degrees of freedom).

**Table S2.** Coefficients (standard errors in parentheses) for the lagged values of the autocorrelation (ACF) and partial autocorrelation (PACF) functions of the residualized value of the incidence of early neonatal deaths (n=126).

| **Lag at month** | **ACF (SE)** | **Ljung-Box**  **Q Statistic**^a^ | **PACF (SE)** |
| --- | --- | --- | --- |
| 1 | -.03 (.08) | .1 | -.03 (.08) |
| 2 | -.05 (.08) | .5 | -.05 (.08) |
| 3 | -.01 (.08) | .5 | -.01 (.08) |
| 4 | -.00 (.08) | .5 | -.01 (.08) |
| 5 | .01 (.08) | .5 | .01 (.08) |
| 6 | -.08 (.08) | 1.5 | -.08 (.08) |
| 7 | -.02 (.08) | 1.6 | -.03 (.08) |
| 8 | .06 (.08) | 2.2 | .06 (.08) |
| 9 | -.12 (.08) | 4.8 | -.13 (.08) |
| 10 | -.00 (.08) | 4.8 | -.00 (.08) |
| 11 | .08 (.08) | 5.9 | .07 (.08) |
| 12 | .13 (.08) | 8.9 | .13 (.08) |

^a^ Q-statistic assesses whether a group of overall autocorrelations differs statistically from 0. None of the Q-statistics reject the null of no difference (based on the chi-square distribution with lag-1 degrees of freedom).

**Figure S1.** Proportion of C-sections by perviable gestational age (22–27 weeks) in California by three time periods between 1989 and 2015.
